# Supplementary material for: Development of SCAR Markers for Genetic Authentication of Metarhizium acridum
Source: J Fungi (Basel). 2024 Apr 4;10(4):269. doi: 10.3390/jof10040269 (PMC11050984; doi:10.3390/jof10040269)
Supplement: Supplementary file 1 [file jof-10-00269-s001.zip › jof-2880673-supplementary/Supplementary Figures S1-S5-27-03-24.pdf]

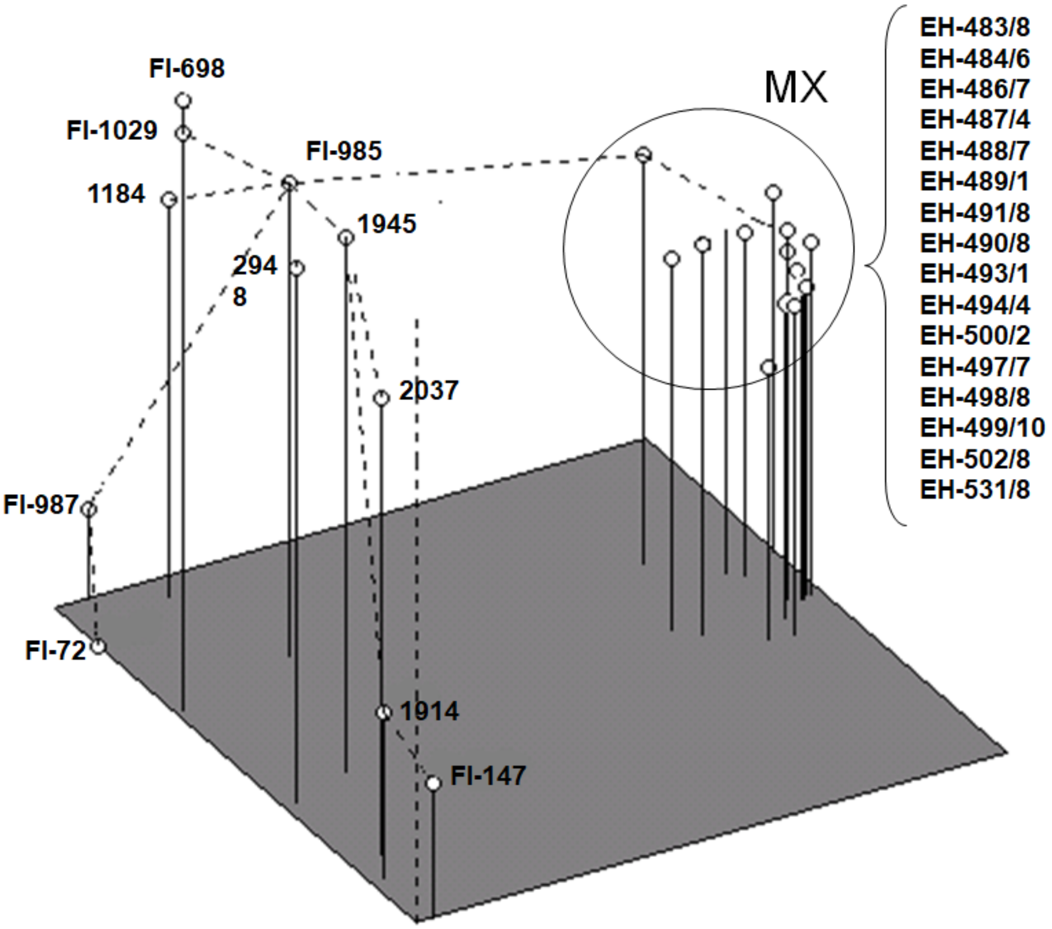

Supplementary Figure S1. Minimum spanning network (MST) and principal coordinates (PcoA) of *M. acridum* DNA. All isolates of MX form one group, directly related to reference strains FI-985 of *M. anisopliae* var. *Acridum* and 1184 of *M. flavoviride* var. *flavoviride*. Isolate/strain identification codes are given in Table 1.

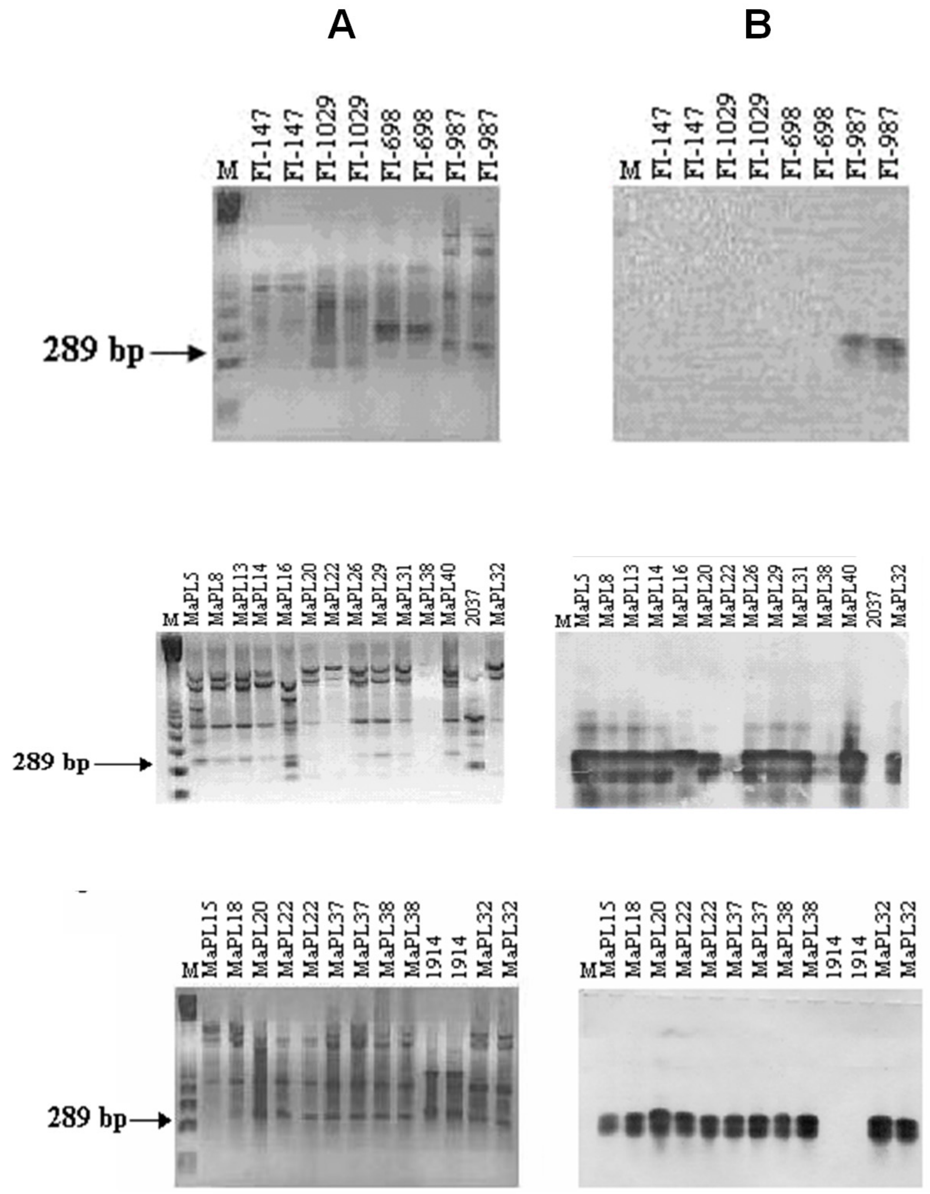

Supplementary Figure S2. Hybridization with the Ma-160OPA-05 probe. A) The figures show the gels with the polymorphic patterns of the *M. acridum* cultures from MX and reference strains. B) The figures show the result of hybridization with the probe.

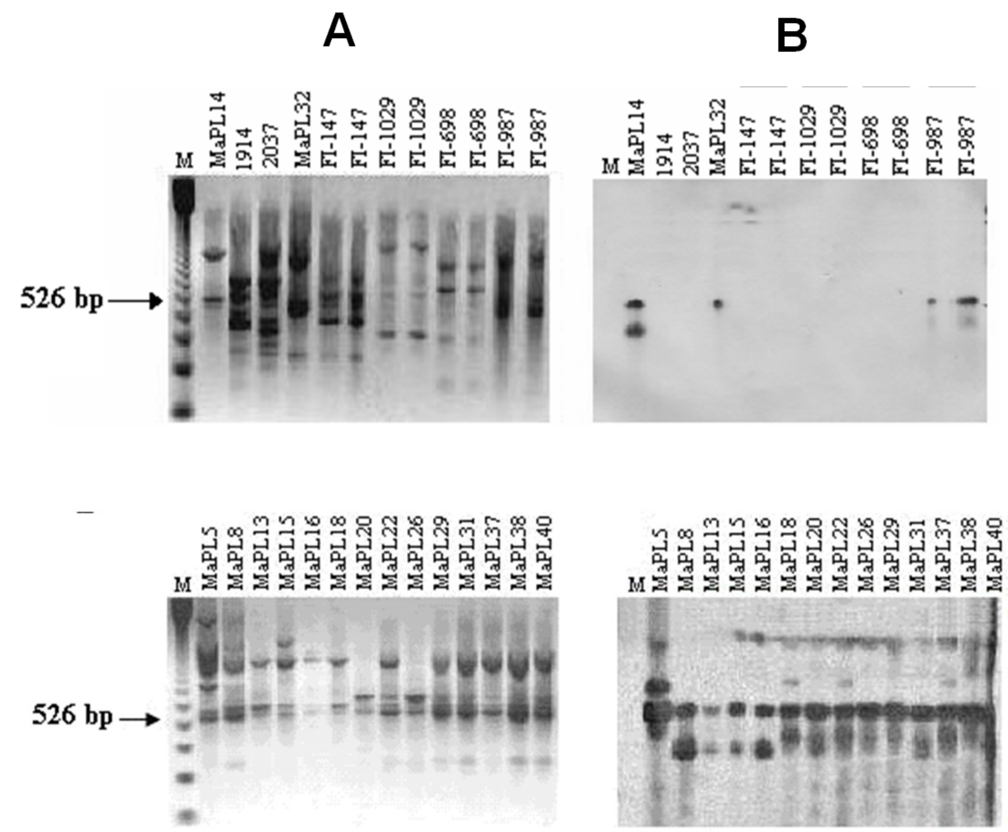

Supplementary Figure S3. Hybridization with the Ma-151$_{OPA-04}$ probe. A) The figures show the gels with the polymorphic patterns of the *M. acridum* cultures from MX and reference strains. B) The figures show the result of hybridization with the probe.

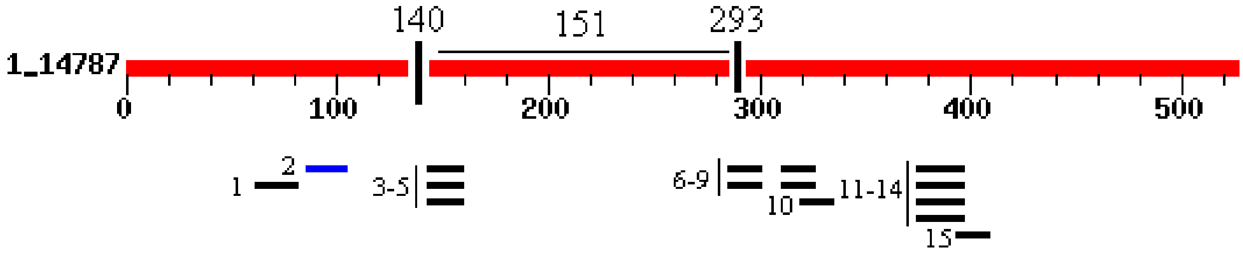

Supplementary Figure S4. Comparison of the clone sequence obtained Ma_{526} with all the sequences registered in GenBank. Black lines indicate regions shared with other fungi: 1 and 15 *Gibberella zea*, 2 and 6-9 *Neurospora crassa*, 3-5 and 11-14 *Saccharomyces cerevisiae* and 10 *Fusarium tricinctum*. The vertical line with 151 indicates the region amplified by the specific oligonucleotides for *M. acridum*.

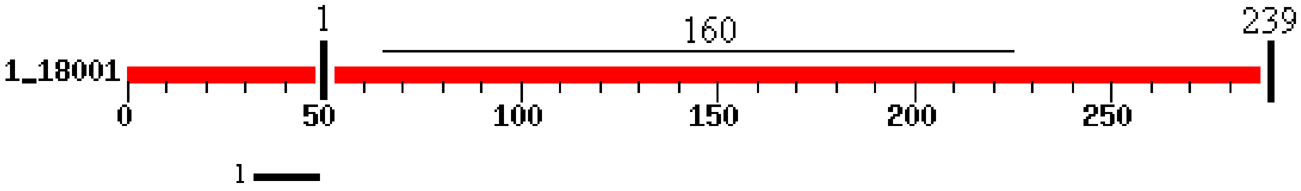

Supplementary Figure S5. Comparison of the clone sequence obtained Ma289 with all the sequences registered in GenBank. The black line indicates the region shared with 1 *Ophiostoma picea*. The vertical line with the number 160 indicates the region amplified by the oligonucleotides specific for *M. acridum.*
